# Internalized stigma among pediatric patients with osteosarcoma and retinoblastoma in Guatemala, Jordan, and Zimbabwe

**DOI:** 10.3389/fonc.2026.1689051

**Published:** 2026-02-06

**Authors:** Anneliese H. Williams, Thelma Velasquez, Inam Chitsike, Hadeel Halalsheh, Ana Cáceres-Serrano, Lucia Fuentes, Nester Chokwenda, Gia Ferrara, Joseph Wardell, Tharwa Bilbeisi, Edith Matsikidze, Nickhill Bhakta, Sima Jeha, Carlos Rodriguez Galindo, Jennifer W. Mack, Victor M. Santana, Sara Malone, Dylan E. Graetz

**Affiliations:** 1 Department of Public Health, Purdue University, West Lafayette, IN, United States; 2 Department of Oncology, Unidad Nacional de Oncología Pediátrica, Guatemala City, Guatemala; 3 Department of Pediatric Oncology, Parirenyatwa Hospital and University of Zimbabwe, Harare, Zimbabwe; 4 King Hussein Cancer Center, Amman, Jordan; 5 Department of Psychology, Unidad Nacional de Oncología Pediátrica, Guatemala City, Guatemala; 6 Department of Global Pediatric Medicine, St. Jude Children’s Research Hospital, Memphis, TN, United States; 7 Department of Oncology, Dana Farber Cancer Institute/Boston Children’s Hospital, Boston, MA, United States; 8 School of Public Health, Washington University in St. Louis, St. Louis, MO, United States

**Keywords:** internalized stigma, global health, pediatric retinoblastoma, pediatric osteosarcoma, ABC model, psychosocial

## Abstract

**Introduction:**

Internalized stigma adversely impacts childhood cancer survivors, limiting their ability to reintegrate with their communities and maintain social networks. However, the impact of internalized stigma on children undergoing cancer treatment is unknown, and no interventions exist to mitigate it. The Activating Events-Beliefs-Consequences (ABC) model from Cognitive Behavioral Theory (CBT) has been used to analyze internalized stigma and inform interventional work. This study employs the ABC model as a conceptual framework to explore how internalized stigma manifests for children recently diagnosed with osteosarcoma and retinoblastoma in Guatemala, Jordan, and Zimbabwe.

**Methods:**

We conducted semi-structured interviews with nine adolescent patients (aged 12-18), 28 caregivers, and 19 clinicians at tertiary cancer centers in Guatemala, Jordan, and Zimbabwe. Interviews occurred in Spanish, Arabic, Shona, and English and were transcribed and translated into English for analysis. Two coders independently coded transcripts, resolving disagreements through consensus and third-party adjudication. A framework analysis used the ABC model to understand manifestations of internalized stigma, defined *a priori* as “a patient’s own adoption of negative societal beliefs or feelings, including changes in self-identification.”

**Results:**

Patients, caregivers, and clinicians all described internalized stigma in pediatric cancer patients. Cancer-related physical changes and community stigma activated internalized stigma. Patients responded to these events by forming beliefs about their appearance, abilities, normalcy, and future, which shaped their behaviors and emotions. While some patients internalized stigmatizing beliefs about themselves and experienced negative consequences, others maintained resilient self-beliefs that fostered adaptive behaviors and emotions. Knowledge about the disease, supportive interactions with survivors and other patients, and caregiver support promoted stigma resilience.

**Discussion:**

Our results demonstrate that internalized stigma impacts pediatric cancer patients from the time of diagnosis, highlighting the relevance of the ABC model in understanding this complex phenomenon. Our findings suggest CBT-based interventions that target patient beliefs, address identified activating events, and enhance multilevel support could help mitigate internalized stigma in pediatric cancer patients. Additional research should explore the efficacy of such interventions, the transferability of our findings to children with other cancer diagnoses and to various geographies, as well as how internalized stigma evolves across the cancer continuum.

## Introduction

Globally, survival rates for childhood cancer continue to rise (1). However, community beliefs surrounding cancer persist, contributing to stigma, a social process in which an individual is labeled, stereotyped, and discriminated against due to an attribute they possess (2). Stigma may follow children with cancer from diagnosis into survivorship (3, 4). Some patients accept community stigmas, leading to internalized stigma, defined as “a stigmatized group member’s own adoption of negative societal beliefs and feelings, as well as the social devaluation, associated with their stigmatized status” (5). In other pediatric illnesses, internalized stigma has been shown to impact patients’ mental health, quality of life, and social functioning (6, 7). While limited stigma research has been conducted in pediatric oncology, a study in South Korea found that pediatric cancer survivors’ internalized shame leads to psychological distress following experiences of stigma (4). Additionally, other work has identified that internalized stigma inhibits young adult cancer survivors from disclosing their diagnosis, limiting their social support and ability to reintegrate with their peers (8, 9).

This research underscores the presence of internalized stigma in childhood cancer patients and highlights its detrimental effects, yet thus far, research characterizing internalized stigma in pediatric oncology has focused nearly exclusively on survivors in high-income settings. Little is known about how this phenomenon affects children who are newly diagnosed or actively undergoing cancer treatment, particularly in diverse global contexts. Accordingly, understanding how internalized stigma manifests in children currently undergoing cancer treatment, including those in diverse settings, is critical for developing effective early interventions.

Models of internalized stigma provide frameworks for understanding how patient exposures to societal stigmas lead to internalized stigma and its negative consequences. By offering insights into this process, these frameworks can guide intervention development. Evidence suggests that cognitive-behavioral models, such as the ABC (“Activating Event-Beliefs-Consequences”) model (10), may be relevant to understanding internalized stigma and informing interventions (11, 12). This model posits that when an individual experiences an upsetting event, their cognitive response shapes the consequences they face. In other words, *activating events* lead individuals to adopt various *beliefs* which, in turn, result in the individual experiencing behavioral and emotional *consequences* that can be either positive or negative. The ABC model has been used in a variety of contexts, including within the designs of stigma and internalized stigma interventions for illnesses such as depression (13), schizophrenia (11), and stroke (14). Its successful use in these areas suggests the model may also be effective in understanding manifestations of internalized stigma in pediatric cancer and shaping interventions for this population.

This study aims to use the ABC model to explore how internalized stigma manifests for children recently diagnosed with cancer in diverse settings to understand its consequences, model the phenomenon, and suggest potential opportunities for intervention.

## Materials and methods

This study is a secondary analysis of a larger study that examined stigma and its impacts on decision-making for pediatric oncology patients with osteosarcoma or retinoblastoma in Guatemala, Jordan, and Zimbabwe (3). Full methods for the original study have been previously reported (3); the methodology relevant to this analysis is described below.

### Setting and participants

Data were collected at three pediatric oncology centers: Unidad Nacional de Oncología Pediátrica (Guatemala), King Hussein Cancer Center (Jordan), and Parirenyatwa Hospital (Zimbabwe).

These three culturally and ethnically distinct sites were chosen to examine cross-cultural commonalities in pediatric cancer stigma. The sites feature different geographies (Latin America, the Middle East, and Sub-Saharan Africa), resource levels (an upper-middle income country, a wealthier lower-middle income country, and a poorer lower-middle income country (15)), and official languages (including Spanish (16), Arabic (17), and Shona/English (18)). Culturally, Guatemala is predominantly Evangelical and Catholic with a diverse Indigenous Mayan population (16). Many Guatemalans perceive cancer as both a physical and spiritual/emotional disease (19) and utilize a combination of Western medicine and traditional healing practices for healthcare (20). On the other hand, most Jordanians practice Islam (17), which conceptualizes illness as predetermined by Allah and emphasizes the importance of accepting his will. Islam encourages the use of medicine but ultimately believes the cure comes from Allah (21). Finally, Zimbabwe is a mostly Christian country with large Shona and Ndebele ethnic populations (18), and its people seek cancer care from traditional healers, Christian faith-healers, and Western medicine (22, 23).

This study focused on the experiences of osteosarcoma and retinoblastoma patients at each center who had been diagnosed within the past 12 weeks. These diseases were chosen because they affect a wide age range of children, and their treatment often involves appearance-altering surgeries. Eligible participants included adolescent patients between ages 12–19 years, caregivers of children with these diagnoses, and their clinicians. Interviews with caregivers and clinicians helped understand how they perceive patients to be impacted by internalized stigma, allowing a deeper understanding of the subject. Potential participants were identified through a review of patient records utilizing purposive sampling, and care teams were informed before patients/caregivers were invited to participate. All participants received a verbal explanation of the study before providing written informed consent in their native languages, and recruitment continued until thematic saturation was reached (24).

Study design was approved by the St. Jude Institutional Review Board and by each site’s human subjects’ protection board.

### Data collection and analysis

Members of the research team trained in qualitative interviewing conducted semi-structured interviews. Questions asked caregiver and patient participants to reflect on their personal experiences with stigma, as well as their opinions about what drives stigma, what stigma practices exist in their communities, what mitigates stigma, and how stigma impacts outcomes for children. The interview guide for providers covered similar topics, but questions were framed to ask about their patients generally rather than the caregiver/patient participant’s personal experience. While the interview guide was designed for the primary study and explored stigma broadly, it prompted discussions of internalized stigma, and some questions asked about the phenomenon directly (e.g. “Has the cancer diagnosis changed the way you [your child] think[s] about yourself [him/herself]?”). The interview guide, which can be found in **Supplementary Material 1**, was developed in English, translated into study languages, and adapted through pilot testing at each study site, with final back translation into English to ensure preservation of initial question intent.

Interviews were conducted in Spanish (Guatemala), Arabic (Jordan), English (Jordan, Zimbabwe), and Shona (Zimbabwe), and were audio-recorded before being transcribed and translated into English by a professional transcription company. Bilingual team members checked transcriptions for accuracy. Four team members with qualitative experience developed the codebook utilizing a combination of *a priori* (deductive) and novel (inductive) codes identified during review and pilot coding of 12 interview transcripts. **Supplementary Material 2** includes the codebook with complete code definitions. After the codebook was finalized, two team members independently applied codes to each transcript; discrepancies were resolved through consensus with third party adjudication. Qualitative data management software MAXQDA 2022 (VERBI Software) (25) facilitated coding and analysis.

This analysis centered on the deductive code “internalized stigma,” defined as: “A patient’s own adoption of negative societal beliefs or feelings, includes changes in self-identification; also includes the opposite which may manifest as confidence or self-esteem.” The ABC model (10) was used as a framework to analyze the relationship between internalized stigma and other coded concepts (including stigma drivers, mitigators, experiences, practices, and outcomes), as well as to identify activating events, beliefs, and consequences relevant to internalized stigma in pediatric cancer patients. Factors mediating the relationships between different parts of the ABC model were explored. Rigor in reporting was ensured using the Consolidated Criteria for Reporting Qualitative Studies (26).

## Results

### Demographics

Across the three sites, nine patients, 28 caregivers, and 19 clinicians were interviewed. Clinicians represented a range of professions including nurses, oncologists, and surgeons. Most caregivers were mothers, although four fathers and one grandparent participated. The average age of patient participants was 15 years, and all but one had a diagnosis of osteosarcoma. The retinoblastoma patient was 12 years old. **Table 1** presents further demographic data for participants.

**Table 1:** Demographics.

Variable	Guatemala	Jordan	Zimbabwe	Total
Patient Variables				
** Number of Participants (n)**	2	5	2	**9**
** Age, years (median)**	13.5 [IQR = 12, 15]	15.75 [IQR = 15, 16.67]	14.5 [IQR = 12, 17]	**15** **[IQR = 13, 16.67]**
Sex (n):				
Male	2	3	1	**6**
Female	–	2	1	**3**
Diagnosis (n):				
Retinoblastoma	–	–	1	**1**
Osteosarcoma	2	5	1	**8**
Extent of Disease (n):				
Localized	2	4	1	**7**
Metastatic	–	1	1	**2**
*Treatment Plan (n):				
Chemotherapy	1	5	1	**7**
Surgery	2	5	2	**9**
Radiation	–	–	1	**1**
Palliation-Only	–	–	1	**1**
Caregiver Variables				
** Number of Participants (n)**	10	10	8	**28**
** Caregiver Age, years (median)**	35.5 [IQR = 30, 38]	40 [IQR = 28, 46]	39.5 [IQR = 34.5, 45]	**37.5** **[IQR = 29.5, 45.5]**
Caregiver’s Sex (n):				
Male	5	6	5	**17**
Female	5	4	3	**11**
** Child’s Age (Median)**	10.29 [IQR = 1.92, 12]	8.5 [IQR = 0.83, 15.75]	6.5 [IQR = 2.5, 9]	**8.5** **[IQR = 1.75, 12]**
Child’s Diagnosis (n):				
Retinoblastoma	5	5	5	**15**
Osteosarcoma	5	5	3	**13**
Child’s Sex (n):				
Male	5	6	5	**16**
Female	5	4	3	**12**
Relationship to Child (n):				
Mother	9	7	7	**23**
Father	1	3	–	**4**
Grandparent	–	–	1	**1**
** Language (n):				
Arabic	–	10	–	**10**
Shona	–	–	8	**8**
Spanish	10	–	–	**10**
Indigenous Language	3	–	–	**3**
Clinician Variables				
**Number of Participants (n)**	8	6	5	**19**
Occupation (n):				
Nurse	2	2	1	**5**
Oncologist/HemOnc Fellow	2	3	–	**5**
Surgeon/Ophthalmologist	2	1	2	**5**
Pediatrician	1	–	1	**2**
Radiation Oncologist	–	–	1	**1**
Social Worker	1	–	–	**1**
Years of Experience (n):				
1–9 years	3	4	4	**11**
10–19 years	3	1	–	**4**
≥20 years	2	1	1	**4**

^*^Patients can receive multiple modalities of treatment, so totals exceed number of patients.

^**^Some caregivers spoke multiple languages, so some totals exceed number of caregivers.

Bold values indicate totals.

### Manifestations of internalized stigma

**Figure 1:**
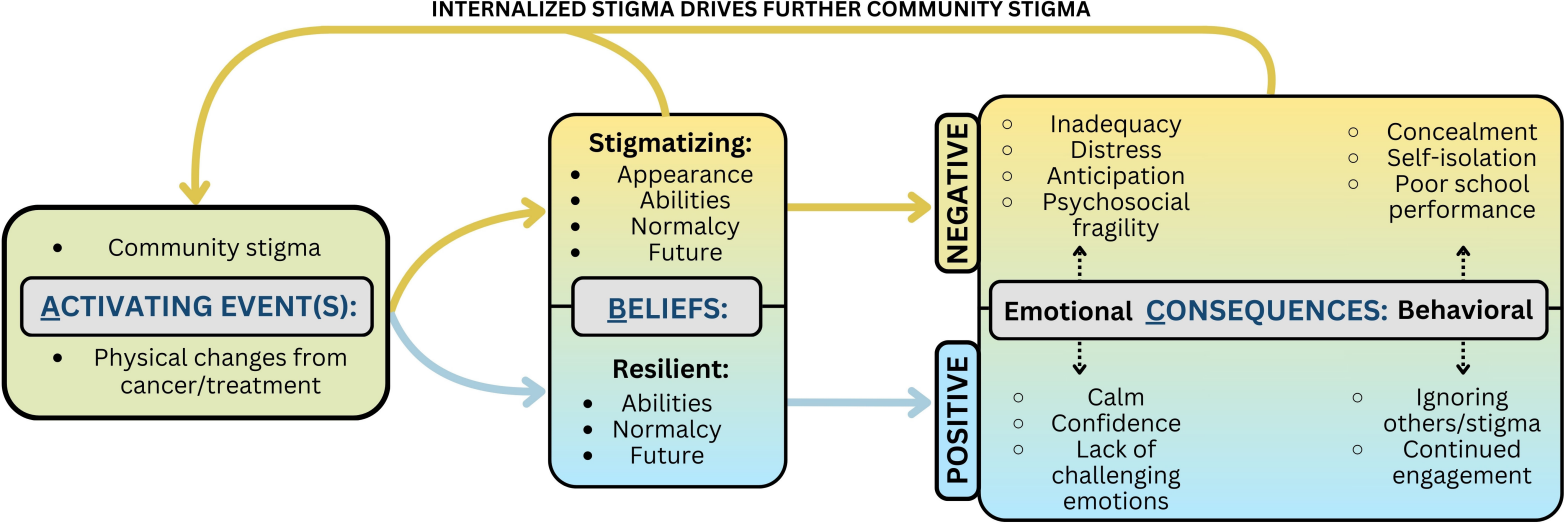
The ABC Model of Internalized Stigma.

Clinicians, caregivers, and patients all acknowledged the presence of internalized stigma in pediatric cancer patients. Various activating events, including physical changes from cancer and its treatment as well as experiences of community stigma, prompted internalized stigma. Internalized stigma in turn resulted in both behavioral and emotional consequences as shown in **Figure 1**.

#### Activating events

A cancer diagnosis activated internalized stigma for patients in two primary ways: through the physical changes that resulted from cancer and its treatment, and through experiences of cancer-related community stigma (**Table 2**). While some participants commented generally on how changes in appearance and ability prompted internalized stigma, others referenced specific aesthetic and functional changes like hair loss, swelling from tumor growth, vision loss, amputation, and use of a prosthesis as activating events. One clinician explained how appearance changes led to internalized stigma, saying: “The appearance may be changed by the treatment. Yeah. And because of that, self-image becomes a problem.” (Ophthalmologist, Zimbabwe). Community stigma, including experiences of bullying, discrimination/exclusion, and prejudice, also activated internalized stigma. For example, a patient from Jordan emphasized how bullying contributed to patients feeling less than others, remarking: “When they see someone who is sick, they bully him and make him feel less than them. They cause him to have mental pressure, or when parents discriminate between their sick child and their healthy one.” (Osteosarcoma patient aged 18), and a Guatemalan caregiver described the impact of comments from family members on a patient’s thought processes, explaining: “so I think that’s what she fears, that they say things that the girl can hear and can affect her, emotionally, lowering her self-esteem.” (Father of osteosarcoma patient).

**Table 2:** Activating event.

Physical changes from cancer and its treatment	
Guatemala	“Teenagers also often try to conceal this, for example in an arm amputation they use long-sleeved shirts, the same with the leg, they try to use a sheet or a towel to hide the physical defect, that leaves a very deep impression on them.” (Pediatric oncology fellow, Guatemala)
Jordan	“You know, having an athlete who enjoys a healthy body suddenly be sick in his knee, it is very difficult for him and he cannot accept these kinds of physical changes. I consider his reaction to being normal.” (Mother of osteosarcoma patient, Jordan) “The other day, his eye got swollen due to the medication, three days later I suggested taking him out for a car ride, but he refused to go saying that he cannot let people see him and that this is only when his eye is swollen, let alone if it was removed and replaced with another eye.” (Father of retinoblastoma patient, Jordan). “So the people think that if they have a disease – well, if they lost vision or lost an eye, they have something deficient in their body.” (Oncologist, Jordan).
Zimbabwe	“But when the eyes start getting bulging and all that creates a situation where it becomes visible that this child has problem. And, obviously, I think people think that - that then it becomes either self-stigma or coming from those that are looking at that person.” (Ophthalmologist, Zimbabwe) “Firstly, my concern was how would my child feel with the amputation? The child might grow up thinking she’s different from other people and so forth.” (Mother of retinoblastoma patient, Zimbabwe)

Community stigma	
Guatemala	“I don’t know, I think it was bad because when they … I never thought there were children with cancer, only older people. And when I looked there at the hospital, a boy came and told me that he had cancer in his little head. And she told me because there was a girl where she lived at the school, and she had cancer. She already died because they couldn’t save her there. And she died and I, when they told me I had cancer, I was scared because I knew that a little boy had cancer, and he died, and I didn’t want to die. Because I want to be with my family.” (Osteosarcoma patient aged 12, Guatemala) “That’s why he [child with an amputated leg] often stays home, he doesn’t come out to avoid being told things” (Osteosarcoma patient aged 15, Guatemala) “Regarding the survivors, we see it even more, because most of them are young and they have the stigma of being a cancer survivor but they cannot work in a company, or they feel less than others, and emotionally they don’t feel capable of working in a company, of living a good life, so it’s very difficult to work with them.” (Social worker, Guatemala)
Jordan	“Family, sometimes they would not let him play with his siblings because of his sickness. They make him feel weak and unable to do what his peers can do. Or they may bring food to this child and not this child because he is sick.” (Mother of retinoblastoma patient, Jordan)
Zimbabwe	No relevant quotations

#### Beliefs

Internalized stigma beliefs centered on four themes: appearance, abilities, normalcy, and the future. For all themes other than appearance, which only arose in the context of internalized stigma, participants described a spectrum of both stigmatizing and resilient beliefs and suggested that patients’ interpretations of cancer-related activating events may change over time. Caregivers and clinicians often perceived patients as holding stigmatizing views about themselves; however, in interviews, patients rarely endorsed these beliefs. Quotes related to each theme of beliefs are listed in **Table 3**.

**Table 3:** Internalized beliefs.

Theme	Stigmatizing beliefs	Resilient beliefs
Appearance	“seeing herself without a leg, she said that as she saw herself how other people were going to look at her, and in the case of the treatment, the chemotherapy, what affected her was the loss of hair, that maybe people would look at her in a weird way … the hat she wore would fall and she would quickly put it back on so people would not see her” (Father of osteosarcoma patient, Guatemala)	No relevant quotations
Abilities	“So sometimes they actually might set the bar low for themselves, because they think they are not capable of they have like a chronic disease that they are being managed for so they are no longer intelligent.” (Pediatric Oncologist, Zimbabwe)	“I have talked with one of my patients- I don’t have a lot of time to do it- but I have talked to one specifically who wants to play sports, and the way he thinks about the disease is to say, I can pull through, even if I have this sickness, or another one, I will still play sports.” (Nurse, Guatemala)
Normalcy/Value	“Yes. They do experience discrimination, but I think part of the discrimination sometimes starts from the cancer patients themselves and their families, because they discriminate themselves from the rest of the people, because they think now, because they have cancer, they are different.” (Pediatric oncologist, Zimbabwe)	“I like to be like my siblings and for my family to treat me as they treat my siblings and not any different. I am not any less than them, I am the same as them.” (Osteosarcoma patient aged 13, Jordan)
Future	“I feel that maybe they are going to humiliate me and all that. I tell my mom, and she says that no one is going to humiliate me. She says to stay calm, that in the future I will have a good job. That’s the only thing that still scares me. I think about what is going to happen in the future, when everything is finished, that’s what I ask myself.” (Osteosarcoma patient aged 15, Guatemala)	“And they always come here to visit them. And they show that they have their prosthesis and walk normally. That’s what encouraged him, and he says mom, I’m going to get on with my life. No, it does not matter. And I know that I am going to continue, and I am going to be cured.” (Mother of osteosarcoma patient, Guatemala)

In caregiver and clinician descriptions, appearance-related stigma beliefs included negative body image, patients viewing their appearance as abnormal, and patients believing others would react negatively to their appearance. Ability-related beliefs included patients viewing themselves as incapable and doubting their ability to play, socialize, and participate in sports. As patients internalized beliefs about their appearance and abilities, they drew conclusions about how these factors would impact their capacity to fit in with their peers/society and lead a normal life. Beyond beliefs that their cancer diagnosis made them different or unusual, some patients thought cancer made them inferior or deficient, which at times manifested as low self-worth and self-esteem. One Jordanian clinician recounted how female patients reacted to the prospect of amputation by adopting interrelated beliefs about their functionality, appearance, and normalcy: “A female patient would question how she will walk with an artificial leg, what kind of life she will lead, how she will live normally and achieve her dreams. She would question her appearance and have doubts about her beauty.” (Nurse). Finally, as patients reflected on their diagnosis, some internalized that it would negatively impact their future either directly (e.g., by impacting their functionality, thereby limiting their future) or indirectly (e.g., by leading to community stigma that would prevent them from reaching their goals). For example, a caregiver described the shift in her son’s beliefs about his ability to attain his career goals, explaining, “Before he said he was going to study to be a policeman. Now he says, “I’m not going to be able to do that, mom, I’m not going to be able to be a policeman” (Mother of osteosarcoma patient, Guatemala). Other participants reflected on the potential impact cancer might have on the patient’s ability to have a family.

Conversely, many patients maintained stigma resilient beliefs after diagnosis, remaining confident in their abilities, continuing to identify with society, and viewing the future as possible. Patients’ stigma resilient beliefs were acknowledged by patients, caregivers, and clinicians. For example, when one patient was asked whether a cancer diagnosis impacted their ideas about themselves, they explained: “No, because I can always play with my brother and with them. And if it is because they are going to give me a foot to walk, and it is always as if you have your foot, and you can always ride your bike, run and stuff.” (Osteosarcoma patient aged 12, Guatemala), emphasizing confidence in their abilities. Patients also described continued identification with their communities and their beliefs that cancer did not make them inferior or change how they viewed themselves. Stigma resilience also included the preservation of self-esteem. Finally, when considering the future, stigma resilient patients adopted beliefs related to cure, life goals, their ability to attend school, and future job prospects: “Has your illness caused you to have a different outlook on your life? Patient: No not yet.” (Osteosarcoma patient aged 17, Zimbabwe).

#### Consequences

Internalized beliefs related to cancer and its treatment shaped patients’ emotional responses. Participants associated the internalization of stigmatizing beliefs with negative emotional consequences, whereas they perceived resilient beliefs to be reflected in positive emotional effects. Participants cited three challenging emotions patients experienced: inadequacy (including feelings of shame, humiliation, and a loss of confidence, often connected to low-self-worth), distress (manifesting as sadness, anger, and discouragement), and anticipation (such as worry and fear, often related to patients’ considerations of how their diagnosis/treatment might impact their future). For some patients, the emotional consequences of internalized stigma extended beyond uncomfortable emotions, manifesting as psychosocial fragility which participants described as the general degradation of mental health, depression, trauma, and personality changes: “It affects them massively causing their mental health to degrade, they change from what they are used to be.” (Osteosarcoma patient aged 18, Jordan). Conversely, some participants suggested the adoption of resilient beliefs mitigated these difficult emotional experiences and identified positive emotional effects of stigma resilience, including calmness, confidence, and a lack of the challenging emotions associated with internalized stigma.

Internalized beliefs similarly influenced patients’ behaviors. Participants associated internalized stigma with three behavioral consequences: concealment, self-isolation, and poor school performance. Participants described how patients attempted to cover or hide visible differences related to cancer and its treatments using sheets, hats, and long sleeves. One clinician explained how patients’ concealment of symptoms at times delayed diagnosis: “On really rare occasions we’ve seen some cases that it was hidden by the patient, and he did not contact anyone because of some … stigma and the patient referred to us in the late diagnosis.” (Surgeon, Jordan). While some patients hid their visible differences in response to internalized beliefs, others avoided social contact. Self-isolation behaviors included patients limiting engagement in activities, refusing to leave the home or have visitors, and resisting being seen in public. Finally, internalized stigma was perceived to affect schooling, leading patients to miss school and negatively impacting their performance: “Emotionally, it affects the way you look at yourself you might lose your self-confidence. So I think and by losing your self-confidence, you don’t perform as well in school or at work” (Oncologist, Zimbabwe). On the other hand, patients’ resilient beliefs encouraged their ability to ignore what people said or thought and to continue engagement in activities. For example, one caregiver explained how their son acknowledged the presence of community stigma but chose to respond by not caring, “and he says that people will criticize you but I don’t care, as long as I eat, and I am okay, people will not mock me, that’s what my boy says,” (Mother of retinoblastoma patient, Guatemala). In Jordan, clinicians also associated stigma resilience with a lack of concealment behaviors and continued engagement in activities.

Additional quotes related to the emotional and behavioral consequences of internalized stigma are presented in **Table 4**.

**Table 4:** Consequences.

Emotions		
Negative	Inadequacy	“For example, when my girl started the treatment, the chemotherapy, her hair began to fall off and she felt ashamed that other people saw her like that and she was affected by that” (Father of osteosarcoma patient, Guatemala)
	Distress	“For instance, there was this boy who was very sad and depressed, he was my patient a few months ago. He was worried about his self-image and his ability to socialize and perform his activities” (Pediatrician, Guatemala) “Other children get angry or depressed, they worry about what is going to happen with them in the future.” (Pediatric oncology fellow, Guatemala)
	Anticipation	“But sometimes it starts with the cancer patients, because they think they are different, I only have a few maybe years or months to live, have this diagnosis that everyone doesn’t want. Sometimes also if they are not – if it’s not explained to them, they might think it’s contagious. Yes. Sometimes they also are afraid maybe if they get into a relationship, got married, have kids, their kids are also going to have something, some cancer. So they are actually afraid. So sometimes they stigmatize themselves, then the community will stigmatize them as well” (Pediatric oncologist, Zimbabwe)
	Psychosocial Fragility	“At the beginning my baby was about to enter into a state of depression, then a child psychologist here at the hospital explained to her what she had, I was with her, because they had told me first, and they told her, and after the first chemotherapy she almost became depressed, she looked at herself, it was a horrible experience for her and for me,” (Mother of osteosarcoma patient, Guatemala)
Positive	Lighter emotions	“Yes, we talk about it, he does pay attention, he ignores it, like he doesn’t hear them and he remains calm” (Mother of retinoblastoma patient, Guatemala)
	Lack of challenging emotions	“Of course, when he meets people who are sick like him, he will feel normal, he will not feel alone or weak. When he sees people like him, he will see himself as a normal child” (Mother of retinoblastoma patient, Jordan) “I don’t know. I’m not ashamed because they took my foot away.” (Osteosarcoma patient aged 12, Guatemala)

Behaviors		
Negative	Concealment	“and the other thing, children with retinoblastoma after enucleation have asymmetric facial features and the parents are always trying to conceal this, they put hats on them or they cover them with a sheet. Teenagers also often try to conceal this, for example in an arm amputation they use long-sleeved shirts, the same with the leg, they try to use a sheet or a towel to hide the physical defect, that leaves a very deep impression on them.” (Pediatric oncology fellow, Guatemala) “It scares me to go out in public like that, I would want to hide it.” (Osteosarcoma patient aged 15, Guatemala)
	Self-Isolation	“On this one, is they themselves – they discriminate themselves from the other groups. Feeling maybe they can’t be loved, and they can be associated with some – I don’t know, because of the ignorance that I talked about before. Yeah, some, they don’t know exactly what the origin of the disease. So fearing or with that fear of including themselves into the community, they will isolate themselves.” (Nurse, Zimbabwe) “My son refused to let anyone visit him because of chemotherapy, he refused to visit people or have anyone ask about him. We were not allowed to talk about him, not even on the phone.” (Mother of osteosarcoma patient, Jordan)
	School Impacts	“I’m expecting that the – how they will feel, they will feel very sad, and they will prefer not to go to school anymore.” (Oncologist, Jordan)
Positive	Ignored Others’ Opinions	“No, I don’t feel like before there, because they scared me there, because they told me that they’re going to cut off your foot, they’re going to cut off your other foot, you’re not going to live, and you’re just going to be very busy with your hand and that. And I didn’t think that, I didn’t pay attention, I just, I just didn’t pay attention, I just let them be.” (Osteosarcoma patient aged 12, Guatemala)
	Continued engagement	“If he feels that, he will be acceptable for the community, and he will get high scores in the secondary school and will go to the university and would get good job. They will say, okay, I will build something big in my life just by being very professional.” (Oncologist, Jordan)

### ABC model

Participants described how manifestations of internalized stigma reinforced each other. Patients responded to activating events by internalizing a spectrum of both stigmatizing and resilient beliefs about themselves and their situation. These beliefs led to emotional and behavioral consequences which, alongside patients’ stigmatizing beliefs, drove further community stigma, restarting the cycle and compounding patient experiences of internalized stigma. An overview of the ABC model as it applies to internalized stigma is depicted in **Figure 1**, and quotes supporting the ABC model relationships can be found in **Table 5**.

**Table 5:** The ABC pathway and mediators.

Activating events → beliefs: patients internalize beliefs in response to activating events 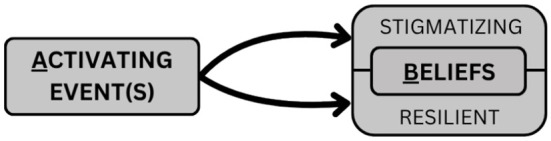	
Activating Events **→** Stigmatizing Beliefs	“Firstly, my concern was how would my child feel with the amputation? The child might grow up thinking she’s different from other people and so forth.” (Mother of retinoblastoma patient, Zimbabwe) “So the people think that if they have a disease – well, if they lost vision or lost an eye, they have something deficient in their body.” (Oncologist, Jordan)
Activating Events **→** Resilient Beliefs	“I have talked with one of my patients- I don’t have a lot of time to do it- but I have talked to one specifically who wants to play sports, and the way he thinks about the disease is to say, I can pull through, even if I have this sickness, or another one, I will still play sports.” (Nurse, Guatemala)
Beliefs → Consequences: internalized beliefs lead to consequences 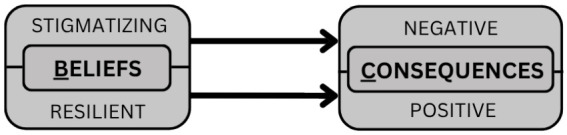	
Stigmatizing Beliefs **→** Negative Consequences	“in the case of the treatment, the chemotherapy, what affected her was the loss of hair, that maybe people would look at her in a weird way, she didn’t like the way she looked, the hat she wore would fall and she would quickly put it back on so people would not see her, so I think both things have an effect related to the stigma” (Father of osteosarcoma patient, Guatemala)
Resilient Beliefs **→** Positive Consequences	“So I think that living with people who have the same disease calms them more and gives them confidence, believe me, because they tell me so. They say, I came back, I talked with someone and they told me that they had had so many surgeries, and I only have had 3, so if they made it this far, why not me? So everybody calms down and the relation is different.” (Nurse, Guatemala)
Beliefs/consequences → community stigma (activating event): stigmatizing beliefs and negative consequences promote further community stigma 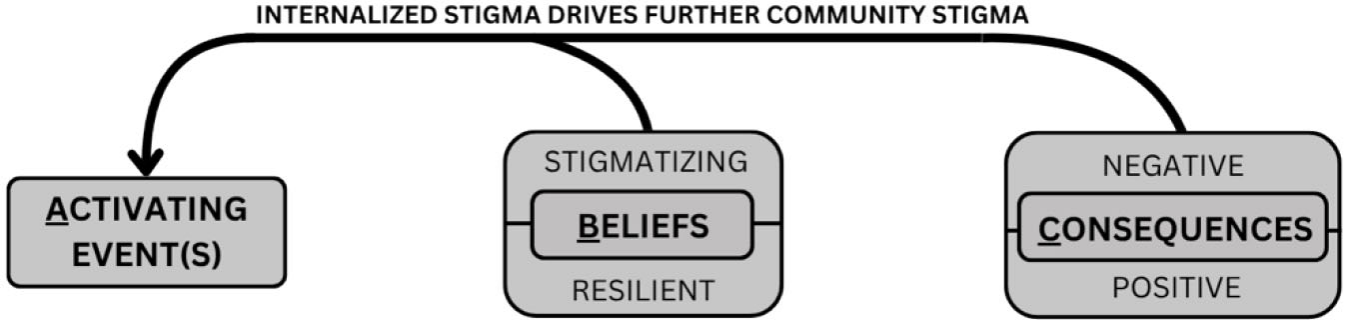	
Stigmatizing Beliefs **→** Community Stigma	“Society will treat you as you want to be treated, if you want to be weak and vulnerable, they will treat you as such. But if you try to merge yourself with them then why not, they will accept you.” (Mother of osteosarcoma patient, Jordan)
Negative Behavioral Consequences **→** Community Stigma	“They do experience discrimination, but I think part of the discrimination sometimes starts from the cancer patients themselves and their families, because they discriminate themselves from the rest of the people, because they think now, because they have cancer, they are different … because I think the exclusion starts from themselves at the beginning. Then the community, sometimes they can then also discriminate or stigmatize them.” (Pediatric oncologist, Zimbabwe)
Mediators: knowledge, interactions with survivors and other patients, and caregiver support mediate internalized stigma 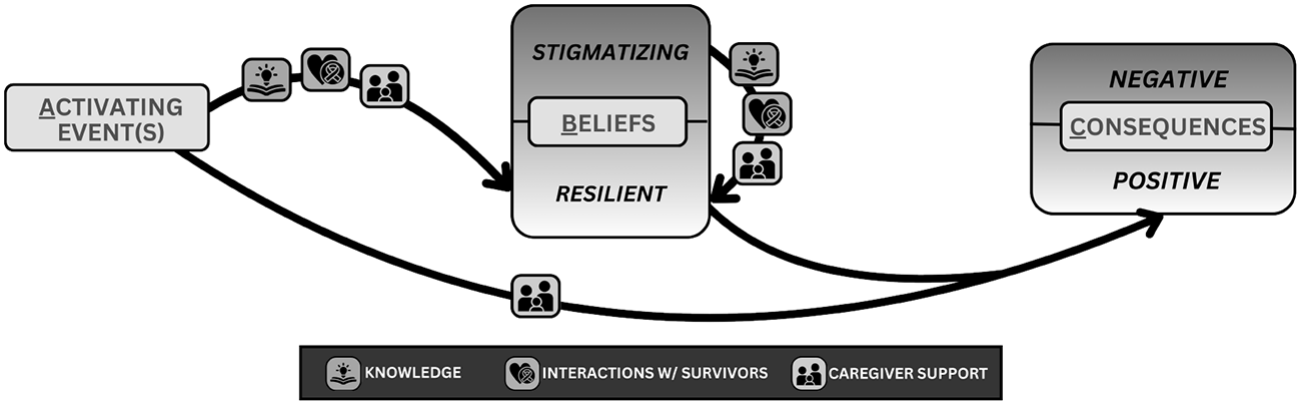	
Knowledge	“The good thing is we try to tell them that most of the sarcomas in the lower limbs they need some they can they can they can be salvaged in other means that they can we can avoid the amputation. And this will might lessen the impact of stigmatization or feeling humiliated by such treatment because the functional status of such being independent after the treatment is something really concerning regarding the family and the patient.” (Surgeon, Jordan) “But sometimes it starts with the cancer patients, because they think they are different, I only have a few maybe years or months to live, have this diagnosis that everyone doesn’t want. Sometimes also if they are not – if it’s not explained to them, they might think it’s contagious. Yes. Sometimes they also are afraid maybe if they get into a relationship, got married, have kids, their kids are also going to have something, some cancer. So they are actually afraid. So sometimes they stigmatize themselves, then the community will stigmatize them as well.” (Pediatric oncologist, Zimbabwe)
Interactions with Survivors and Other Patients	“For instance, there was this boy who was very sad and depressed, he was my patient a few months ago. He was worried about his self-image and his ability to socialize and perform his activities, and I remember there is a young man in social media who speaks about this, that he had cancer, I guess osteosarcoma because he is an amputee, and he gives motivational talks, my patient sees these videos and he appears doing exercises, he looks very well, and my patient told me that he will overcome it as well, that helped change his mind and to accept it.” (Pediatrician, Guatemala) “They always come here to visit them. And they show that they have their prosthesis and walk normally. That’s what encouraged him, and he says mom, I’m going to get on with my life. No, it does not matter. And I know that I am going to continue, and I am going to be cured.” (Mother of osteosarcoma patient, Guatemala)
Caregiver Support	“He told the girl who was passing handicrafts, he told to the young lady: “I’m not going to be able to play anymore” and she told him: “well, you’re going to be able to play … But we need to talk to him a little bit to tell him that things can be done, especially more information to talk to him to tell him that things can be done and now he is calm because I talk to him and I tell him: “My son, look you are going to walk.” I tell him: maybe you are not going to be able to do the same as before, but you are going to walk, you are going to stand up, you are going to put on your pants”, I tell him.” (Mother of osteosarcoma patient, Guatemala) “That is why we need to motivate our children and make them feel normal. We need to motivate them and give them self-confidence so that they can face whatever comes at them.” (Mother of retinoblastoma patient, Jordan). “If there is no enough family support and if the child, for example, went to school and encountered bullying, so he will just lose his self-confidence,…So that’s why I think it’s very important to have a very supportive family, because if the family is supportive, they will just give the needed confidence to the child” (Oncologist, Jordan).

### Mediators of the ABC pathway

**Figure 2:**
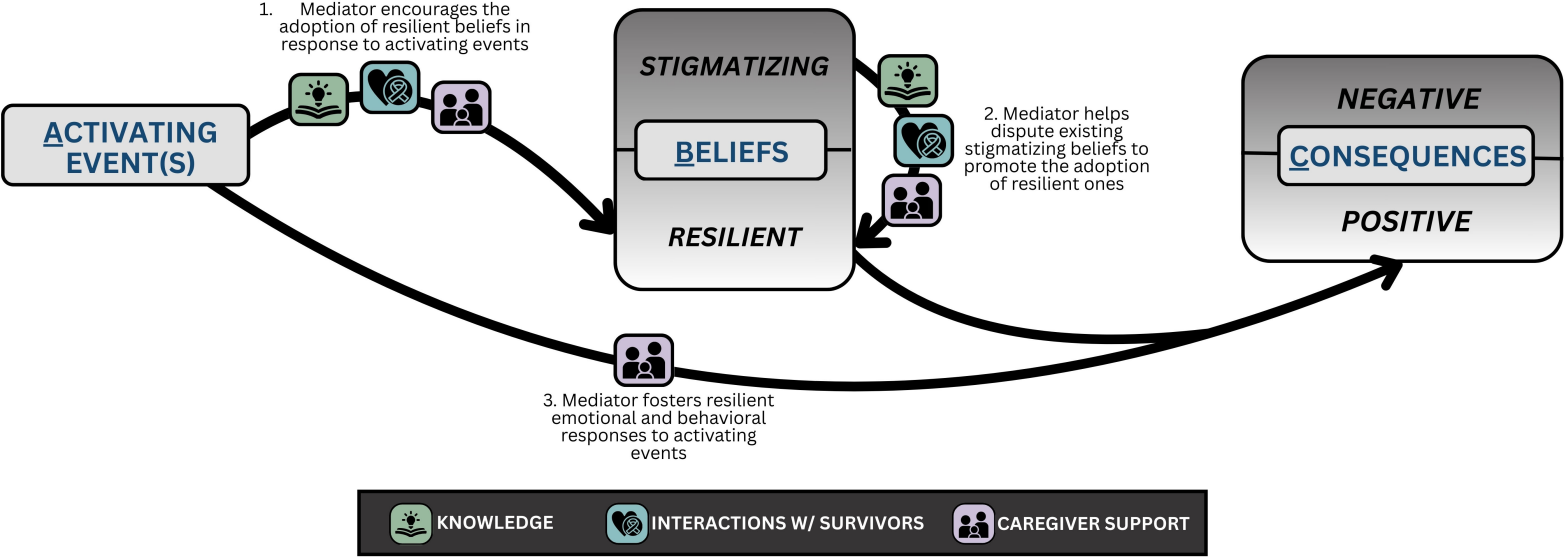
Mediators of the ABC Pathway of Internalized Stigma.

Knowledge, interactions with other patients and survivors, and caregiver support shaped how patients responded to activating events, mediating the ABC pathway of internalized stigma. As described by participants and illustrated in **Figure 2**, these mediators interacted with the ABC pathway at three points: by encouraging the adoption of resilient beliefs in response to activating events, by disputing existing stigmatizing beliefs to promote the adoption of resilient beliefs, and by fostering resilient emotional and behavioral responses to activating events.

Lack of basic knowledge about cancer (such as knowledge about who it impacts and the possibility of survival) and cancer misconceptions contributed to patients’ negative beliefs. On the other hand, anticipatory guidance helped patients adopt a realistic understanding of their diagnosis and treatment, mitigating internalized stigma. Participants cited specific information that was helpful, including information on the availability of prosthesis, the necessity of treatment, and the potential for normalcy in the future. For example, one Guatemalan patient highlighted the protective effect of knowledge about the possibility of cure when they encountered community stigma, explaining: “They just tried to scare me and I didn’t pay attention, because I already knew, I don’t care that they were going to cut my leg. Because I know everything, and that’s how I’m going to live” (Osteosarcoma patient aged 12, Guatemala). Clinicians and caregivers also reflected on how interactions with other patients and survivors, including direct contact with them and viewing their social media posts/videos, cultivated an understanding that the future was possible and fostered a sense of normalcy. One Jordanian caregiver described the impact of meeting other patients on her son’s beliefs about his normalcy and emotional state, explaining: “When he meets people who are sick like him, he will feel normal, he will not feel alone or weak. When he sees people like him, he will see himself as a normal child.” (Mother of retinoblastoma patient). Finally, caregivers expressed their perception of their own impact on their children’s mindsets and understanding of cancer, including how they built confidence and supported resilience. Caregivers reinforced knowledge about cancer and its treatment and provided patients reassurance after experiences of community stigma. Moreover, some participants, including clinicians, suggested a lack of caregiver support left patients vulnerable to internalized stigma.

### Internalized stigma across sites

Internalized stigma and stigma resilience were described similarly by participants from Guatemala and Jordan. However, Zimbabwean participants did not discuss internalized stigma as often. In Zimbabwe, references to internalized stigma were made almost exclusively by clinicians, and only one reference to stigma resilience was made. Furthermore, no Zimbabwean participants identified community stigma as an activating event, and knowledge was the only mediator of internalized stigma they mentioned.

## Discussion

For many diseases, internalized stigma is understood to have pervasive impacts on patients’ social functioning (27, 28), psychosocial health (28–30), and wellbeing (28, 31). This study extends previous work on internalized stigma (32, 33) to highlight the impacts of internalized stigma on pediatric oncology patients from the time of diagnosis, which has important implications on care access and decision-making. Internalized stigma associated with physical changes related to cancer may lead patients to conceal symptoms and avoid help-seeking, thereby delaying diagnosis. At diagnosis, internalized stigma may shape decision-making, contributing to treatment abandonment.

Utilizing the ABC model to analyze internalized stigma in childhood cancer patients across three diverse settings enabled identification of activating events (physical changes and community stigma), internalized beliefs (about appearance, abilities, normalcy, and future) and consequences (emotional and behavioral), and operationalization of the process by which patients adopt stigmatizing beliefs about themselves. While the model appears globally applicable, differences in how participants from each country discussed internalized stigma suggest some aspects may be contextually or culturally specific. This finding is consistent with previous work in childhood cancer that indicates that although many aspects of community stigma are shared between countries, there are nuanced differences in how stigma presents across cultures (3). Cultural dimensions, including collectivism (34, 35) and religious beliefs (36, 37), are known to impact experiences of internalized stigma and may have contributed to site-level differences in our findings. Additional cultural factors such as disclosure discomfort and societal views of illness could also have impacted how participants at each site discussed internalized stigma.

Although internalized stigma was referenced by all types of participants, clinicians’, caregivers’, and patients’ perspectives differed. Clinicians and caregivers similarly acknowledged a spectrum of both internalized stigma and stigma resilience in patients, describing some children as heavily impacted by internalized stigma, and others as remaining resilient. Notably, although some interviewed adolescents referenced other patients’ internalized stigma, they rarely endorsed being affected by internalized stigma themselves and instead focused on their own stigma resilience. Patients’ limited acknowledgement of their own internalized stigma leaves the question of whether they truly experience as much internalized stigma as clinicians and caregivers perceive. One possibility is that patients do internalize stigma but find it challenging to discuss during in-depth interviews. This hypothesis is supported by another component of the same study, in which these patients endorsed internalized stigma on quantitative stigma scales (38), suggesting they do experience it and may find it easier to disclose through a survey. Various factors could impact patients’ discussion of internalized stigma during qualitative interviews. Patients may not be fully aware of their internalized stigma, or they may be aware of it but unwilling to talk about it, which could itself be a manifestation of internalized stigma. Feelings of shame may make patients hesitant to acknowledge their own internalized stigma but more willing to discuss other patients’ experiences as a form of indirect disclosure. Furthermore, research suggests that stigmatized individuals selectively compare themselves to others that have the same stigmatized identity but experience worse outcomes as a way to protect their self-worth and build their own resilience (39). Thus, patients’ limited acknowledgment of internalized stigma might reflect a protective coping mechanism. Other aspects of our study design may also have caused us to miss patients who are suffering from internalized stigma. Namely, many more interviews were conducted with clinicians and caregivers than with patients, which alongside the use of purposive sampling and few interview guide questions asking directly about internalized stigma, may have limited patient references to this phenomenon in our results.

Conversely, it is possible that patients are more stigma resilient than their caregivers and clinicians perceive. Caregivers and clinicians have been noted on quantitative measures of stigma experiences to be poor proxy reporters (38) and have difficulty distinguishing their own beliefs and experiences from patients’. The detailed reflection encouraged by in-depth interviews may make distinguishing these even more challenging. Community cancer stigma may also lead adults to project their own beliefs onto patients and assume patients experience more internalized stigma than they do. Similarly, associative stigma experienced by caregivers of children with cancer (3) could cause them to conflate their children’s response to stigma with their own, complicating their ability to accurately describe their children’s internalized stigma. Ultimately, a combination of these factors is likely at play: patients internalized shame may shape if and how they disclose their own internalized stigma, and the unique experiences of patients, caregivers, and clinicians likely lead them each to perceive patients’ internalized stigma differently.

It is important to consider the discordance between patient, caregiver, and clinician perceptions of internalized stigma when measuring and addressing internalized stigma. As studies work to better characterize the extent of internalized stigma impacting pediatric oncology patients globally, they should clearly delineate between their use of self-report and proxy measures and should avoid using these measures interchangeably in analyses. Moreover, given the complex factors influencing self- and proxy-reports of internalized stigma, researchers should consider using mixed method assessment tools that contextualize quantitative results with qualitative data when appraising internalized stigma. While caregivers and clinicians may have valuable insights regarding intervention design, patients must also be directly involved in intervention design processes to ensure resultant interventions reflect and address children’s actual experiences of internalized stigma.

Given the extent of potential harm associated with inaction if patients are truly experiencing internalized stigma, interventional work targeting the cycle of internalized stigma should be conducted in parallel with studies that further explore the prevalence of internalized stigma in this population. All types of participants in this study described detrimental and pervasive impacts of internalized stigma on pediatric cancer patients, including socioemotional and behavioral consequences. Currently, interventional work targeting internalized stigma in pediatric patients is limited, and no interventions specific to stigma in pediatric cancer patients have been published (40).

Descriptions from caregivers and clinicians suggest pediatric cancer patients’ individual experiences of internalized stigma are heavily influenced by interpersonal factors, and thus, future interventions should target multiple ecological layers. By identifying multilevel components of patients’ internalized stigma, the ABC model highlights potential targets for intervention. At the individual level, interventions should build patient knowledge, help reframe stigmatizing beliefs, and encourage resilient behaviors, using principles of Cognitive Behavioral Therapy (41) which focuses on the relationships between events, cognitive appraisals, and emotions/behaviors. For example, while physical changes from cancer treatment may be unavoidable, particularly in resource-limited settings where limb salvage surgery may not be available or affordable (42), interventions can shape patients’ cognitive responses to these changes; psychologists could facilitate CBT-based exercises (43, 44) that incorporate psychoeducation about anticipated physical changes and ways to cope with them, cognitive restructuring of patients’ beliefs about themselves and their cancer, goal setting, and role playing to help children develop adaptive cognitive and behavioral responses to these changes. At the interpersonal level, interventions should foster opportunities for social interaction with survivors early in treatment and cultivate supportive interactions from caregivers. Although more work is needed to explore the utility of such interventions in pediatric oncology populations, peer support groups (45) and support groups for parents (46) have had promising results for youth with epilepsy in low- and middle-income countries. Such interventions require facilitator training but minimal additional resources to implement, enhancing their feasibility in resource-limited settings. Finally, at the community level, interventions must address community stigma, one identified driver of internalized stigma. Although historically internalized stigma has not been measured as an outcome variable of community-level stigma interventions, likely because it is a distal outcome, our results suggest it is relevant to these interventions.

Our findings come from interview data with patients, caregivers, and clinicians in three settings, representing two diagnoses. While the use of qualitative interviews provided a rich understanding of how patients in these settings experience internalized stigma, to understand whether these findings are transferable to other contexts, future internalized stigma work should be conducted in other settings and with more patients, including patients across age groups, those with other diagnoses, and additional children with retinoblastoma. In addition to the potential lack of thematic saturation for patient perspectives described earlier, translation of interviews into English for analysis could have impacted findings. Finally, given that this study centered on the experiences of children within 12 weeks of diagnosis, longitudinal research should build on these findings to describe internalized stigma in patients across the cancer continuum and analyze how internalized stigma may change from diagnosis, through treatment, and into survivorship. Such future work should include further detailed qualitative exploration of how internalized stigma manifests as well as quantitative work to characterize the extent of internalized stigma experienced by patients.

This study adds understanding of the influence of culture on experiences of internalized stigma for children with cancer, modeling the process by which newly diagnosed patients react to activating events by internalizing stigma and identifying actionable targets for intervention. Although future work is needed to better understand patient perspectives on internalized stigma, our results suggest that interventional work should not be delayed. Our findings can be used to inform this work to encourage stigma resilience and promote positive outcomes for cancer patients.

## Data Availability

The raw data supporting the conclusions of this article will be made available by the authors, without undue reservation.
