# Supplementary material for: Internalized stigma among pediatric patients with osteosarcoma and retinoblastoma in Guatemala, Jordan, and Zimbabwe
Source: Front Oncol. 2026 Feb 6;16:1689051. doi: 10.3389/fonc.2026.1689051 (PMC12920227; doi:10.3389/fonc.2026.1689051)
Supplement: Supplementary file 1 [file Table1.docx]

**SUPPLEMENTARY MATERIAL 1: Interview guide**

**1.**

**1a. [TO BE ASKED DURING INTERVIEWS WITH PATIENTS AND PARENTS] Tell me about your experience finding out you [your child] had cancer.**

1. How did you know about this hospital?
2. What made you come in?
3. Had you heard about cancer before you arrived?

**1b. [TO BE ASKED DURING INTERVIEWS WITH HEALTHCARE PROVIDERS] Tell me about how the children and families you care for usually find out they have cancer.**

1. Do most families know about the diagnosis before they arrive here?
2. Who talks to them once they arrive? And what is said?

**2. Some people feel that there may be stigma (*shame, dishonor, discrimination, humiliation)* associated with a cancer diagnosis. What do you think about this?**

1. Do you/your child/your family/your patients experience stigma related to the diagnosis of cancer?

**3. What do you think drives or contributes to stigma?**

1. Are there factors related to the disease? *(address concealability, disruptiveness, aesthetic qualities, origin, peril)*
2. Are there factors specific to you/your family/your patients? (e.g. education level, religious beliefs)
3. Are their factors related to you/your family/your patients’ community? (e.g. cultural norms)
4. Do you/your child/your family/your patient have fears or worries about the future related to stigma?
5. What about concerns about where the disease might have come from or what caused it?
6. How do you/your child/your patient feel about the surgery that will be a part of his/her/your cancer treatment?

**4. Are there any things that help alleviate or lessen the stigma you/your family/your patients feel?**

1. Did you/your family/your patient know anyone else with a similar diagnosis? If so, did this help?
2. Was there anything you were told at the cancer center [*for healthcare providers*: is there anything you say to families] that helps?
3. Was there anything about the disease that helps lessen stigma? *(address concealability, disruptiveness, aesthetic qualities, origin, peril)*
4. Was there anything that specifically helps you/your child/your family/your patients in relation to the surgery?

**5. [ONLY TO BE ASKED IN PATIENT/PARENT INTERVIEWS] Have you/your child/your family experienced any form of stigma or discrimination since being diagnosed with cancer?**

1. Has the cancer diagnosis changed the way you [your child] think[s] about yourself [him/herself]?
2. Has the cancer diagnosis changed the way people in your community think about you/your child/your family?  Or the way people treat you/your family/your child?
3. Are you worried that you/your family/your child might experience discrimination in the future?

**6. What type of stigma practices exist in your community/ the communities you treat?**

1. Are there common stereotypes about people diagnosed with cancer?
2. Are cancer patients or their families excluded or isolated in any way?
3. Do cancer patients and their families experience discrimination?

**7. How do you think stigma affects outcomes for children with cancer?**

1. Does it affect you/your family/your patient’s willingness to accept or continue treatment?
2. Is this different when you think about chemotherapy compared to surgery or radiation?
3. Has it affected you/your family/your patient’s quality of life?

**8. What are things that you think could be done to lessen stigma or its impact on pediatric cancer care?**

1. Are there things that could be done by healthcare providers at the cancer center?
2. Are there things that could be done at the level of the patient/family?
3. Are there things that could be done in the community?
