# Supplementary material for: Internalized stigma among pediatric patients with osteosarcoma and retinoblastoma in Guatemala, Jordan, and Zimbabwe
Source: Front Oncol. 2026 Feb 6;16:1689051. doi: 10.3389/fonc.2026.1689051 (PMC12920227; doi:10.3389/fonc.2026.1689051)
Supplement: Supplementary file 2 [file Table2.docx]

| **Supplementary Material 2: Code Names and Definitions** | |
| --- | --- |
| **Code Name** | **Code Definition** |
| *Drivers* | |
| Functionality | Inability to walk, see, work or return to school; to be used for references to present time period |
| Fear of future social and economic ramifications | Concerns about ability to work or participate in social life (e.g. marriage) in the future |
| Myths | Misconceptions or community beliefs around cancer |
| Aesthetic qualities | Extent to which cancer is visible to others and/or visibility of cancer leads to stigma marking or a disgust reaction. Includes references to attempts to conceal disease |
| *Mitigators* | |
| Support: Survivors | Support from others with a similar experience, may be cancer or amputation/blindness for another reason. Often found within the hospital (e.g. support groups on WhatsApp) but not exclusively. May include survivors, or current patient/families. Includes references to large foundations/associations for patients or survivors with a particular diagnosis |
| Support: Parents/Family | Parent or family member support for patient |
| Knowledge | Information, or understanding about cancer and the treatment process, may come many sources including personal experience, communication with the medical team, awareness of other's experiences, and the media. Includes parent/patient/family knowledge as well as knowledge of healthcare team. Also includes lack of knowledge or ignorance. Different from uncertainty in that it refers to things that could be known but are not. Does NOT include references to various sources of information without inclusion of information being shared. |
| Prior experiences | With cancer or with amputation/blindness |
| Patient/Family Characteristics | Includes demographics such as education, literacy, primary language of family as well as child's personality, age, gender, type of disease |
| Normalcy | Sense of being the same as someone without cancer; societal acceptance and "fitting in", including maintenance of activities of daily living including ability to go to school/work, looking normal, and participating in usual activities. Also includes the lack of normalcy as it relates to society. |
| *Stigma Experiences* | |
| Anticipated stigma | Concern about future stigmatization, may be double coded with driver |
| Self-stigma | Internalized stigma or a patient’s own adoption of negative societal beliefs or feelings, includes changes in self-identification; also includes opposite which may manifest as confidence or self-esteem |
| Emotional experience of stigma | Sadness, fear, grief, worry, anxiety, shame experienced as a result of stigma |
| Isolation | Loneliness, exclusion from previous activities, seclusion at home, done by/the choice of the patient/family |
| *Stigma practices* | |
| Stereotypes/Prejudice | Beliefs about characteristics associated with the group and its members that may contribute to negative evaluation of the group and its members |
| Stigmatizing behavior | Exclusion from social events, avoidance behaviors; inflicted upon the child/parent; includes discrimination and discriminatory attitudes or belief that people with a specific health condition should not be allowed to participate fully in society |
| Bullying/Humiliation | Teasing, making fun of; public bad acts directed at patient due to cancer/treatment effects |
| *Outcomes* | |
| Adherence | Continuation or ultimate acceptance of therapy |
| Resilience | Ability of patient/family to find strength despite/because of stigma, includes perseverance |
| Abandonment | Upfront refusal of therapy or >4 weeks without cancer directed therapy |
| Psychosocial fragility | Poor mental health, opposite of resilience |
| Delayed diagnosis | Late presentation to care including lack of presentation at the time of original symptom onset, also includes barriers leading to delays reaching the cancer center |
